# The Relationship between Phytochemical Composition and Biological Activities of Differently Pigmented Varieties of Berry Fruits; Comparison between Embedded in Food Matrix and Isolated Anthocyanins

**DOI:** 10.3390/foods8120646

**Published:** 2019-12-05

**Authors:** Izabela Koss-Mikołajczyk, Barbara Kusznierewicz, Agnieszka Bartoszek

**Affiliations:** Department of Food Chemistry, Technology and Biotechnology, Gdańsk University of Technology, 11/12 Narutowicza St., 80-233 Gdańsk, Polandbarbara.kusznierewicz@pg.edu.pl (B.K.)

**Keywords:** anthocyanins, antioxidants, cytotoxicity, detoxification, diabetes, chemoprevention

## Abstract

The aim of this research was to correlate the composition of phenolic compounds and bioactivities (antioxidant, cytotoxic, antigenotoxic, and influence on selected enzymatic activities) exhibited by extracts from differently pigmented raspberry (yellow and red), grape (white and red), mulberry (white and black), and currant (white, red, and black) varieties. It was presumed that phytocomplexes of the same species will be similar while differing significantly only by the content of anthocyanins in colored varieties, which will impact biological properties. Additionally, to compare food matrix embedded and isolated anthocyanins, the influence of purified solution, in concentrations matching the total concentration of anthocyanins in appropriate colored plant samples, on investigated biological activities was studied. The phenolic compound composition and antioxidant profiles showed that anthocyanin content was correlated only with antioxidant capacity of tested plant extracts. The other determined biological activities failed to reveal any simple relationship between chemopreventive potential and anthocyanin composition in fruits studied nor any similarity to biological properties of isolated cyanidin-3-*O*-glucoside. These observations indirectly support the food synergy concept, that points to interactions between different phytochemicals as a factor deciding about the final bioactivity of edible plants.

## 1. Introduction

Discovery of the crucial role of unbalanced redox homeostasis (commonly known as oxidative stress) in etiology of noncommunicable diseases (type 2 diabetes, cardiovascular disease, or cancer) [1], stimulated the interest in antioxidant phytochemicals. Those with documented chemopreventive properties, such as antithrombotic [2] anticarcinogenic [3,4], antidiabetic [5], or antiobesity [6] activities were of special interest. In consequence, edible plants (fruits, vegetables, and herbs) that are rich sources of bioactive compounds are thus by analogy expected to exhibit corresponding health-promoting properties. Furthermore, colored varieties of fruits and vegetables are believed to be characterized by stronger chemopreventive potential than their colorless counterparts, because of the presence of bioactive pigments such as anthocyanins, betalains, and carotenoids that show particularly strong antioxidant potential. However, pigments are not the only antioxidant phytochemicals, though they indeed frequently show stronger antioxidant potential than other redox active compounds (e.g., phenolic acids, flavan-3-ols, etc.) [7,8,9]. 

The relationship between chemical composition and biological activity of differently pigmented plant varieties has not been a widely studied subject in food science. Even if undertaken, these studies usually are aimed at comparison of plant material and limited to the determination of bioactive phytochemical composition and antioxidant potential [10,11,12,13,14,15,16,17]. Generally, in the case of studies comparing differently pigmented varieties of edible plants, three research approaches can be found. The first approach is purely analytical. The focus is mainly on the composition of bioactive compounds such as carotenoids, betalains, or anthocyanins and other polyphenols. Occasionally, these studies have been extended to the analysis of the content and composition of various nutrients (proteins, lipids, saccharides, water, minerals, etc.) in e.g., differently pigmented loquats [14], prickly pears [7], currants [10], or mulberries [12]. The second approach embraces combination of chemical analysis with the assessment of antioxidant potential. Sumaya-Martinez et al. [15] in their paper on prickly pear (*Opuntia ficus-indica*) analyzed not only composition of betalains and phenolic compounds, but they also determined the content of ascorbic acid and antiradical activity (using DPPH—1-diphenyl-2-picrylhydrazyl radical assay and FRAP—ferric reducing antioxidant potential test), and protection against oxidation of β-carotene-linoleic acid emulsion). In the study of Sanchez-Sulcedo et al. [16] on white and black mulberries (*Morus* L.), besides the composition of phenolic compounds, researchers also assessed the content of protein and minerals and antioxidant capacity (using FC—Folin–Ciocalteu’s and DPPH assays) of tested plant varieties. Matsafiji et al. [13] analyzed the content of carotenoids, tocopherols, sugars, organic acids, and antioxidant capacity (DPPH) of white, green, orange, and red chilli pepper (*Capsicum*). The third approach is the most complex one. It involves not only comparison of the composition of bioactive phytochemicals, nutrients, and antioxidant capacity of tested plant varieties, but also their biological potential [9,17,18,19]. For instance, Kim et al. [20] studied antioxidant and antiproliferative properties of polyphenols in white and red pitaya. Garcia-Lafuente et al. [18] described the dependence of antioxidant as well as anti-inflammatory activity of extracts from white and red beans on the content of phenolic compounds.

In this study, we propose alternative exploitation of differently colored fruit varieties, i.e., as indicators of chemopreventive potential of anthocyanin when still embedded in fruit matrix. The relationship between composition of phenolic compounds and health-promoting potential was examined with the use of differently pigmented varieties of berry fruits, namely raspberries (yellow and red), grapes (white and red), mulberries (white and black), and currants (white, red, black). We assumed that chemical composition of the same plant species will be similar and that colored varieties will be distinguished mainly by the presence of anthocyanins. Should these pigments influence chemopreventive properties, this would be reflected by the differences in biological activity. Although isolated anthocyanins are documented to display various biological properties, little is known if their activity is modulated by the presence of other plant matrix components. Our study was aimed at shedding some light on the currently not satisfactorily explained epidemiological observations, that whole fruits and vegetables are more efficient in preventing noncommunicable diseases than bioactive compounds isolated from them. 

To achieve this goal, we characterized the composition of phenolic compounds in tested fruit extracts by HPLC-DAD-MS along with the profiles of ABTS-reactive antioxidants by HPLC coupled with post-column derivatization and antioxidant capacity by standard spectrophotometric tests (ABTS, DPPH, FC, and FRAP) as well as by Cellular Antioxidant Activity (CAA) assay. Biological activity of tested plant extracts was assessed by their impact on the cell viability (MTT - thiazolyl blue tetrazolium bromide test), ability to protect DNA against oxidative damage (comet assay), on the induction of detoxification enzymes, and inhibition of glycoside hydrolases. Based on the results of these determinations and comparison with results obtained for purified cyanidin-3-*O*-glucoside, the attempt to define the relationship between the composition of phenolic compounds and chemopreventive potential of tested berry fruits was undertaken. By comparing experimental results of various biological activities for studied fruit extracts and purified anthocyanin, we tried to pinpoint the properties that result specifically from the presence of tested pigments, and those that just reflect the influence of plant metabolome. Such a relationship, to the best of our knowledge, has not been investigated in as much detail so far.

## 2. Materials and Methods 

### 2.1. Chemicals and Reagents

The following chemicals and biochemicals were used: formic acid, acetonitrile, sodium chloride, hydrochloric acid, sodium hydroxide, hydrogen peroxide (H_2_O_2_), 2,2-azinobis-(ethyl-2,3-dihydrobenzothiazoline-6-sulphonic acid) diammonium salt (ABTS^+^), 1-diphenyl-2-picrylhydrazyl (DPPH), thiazolyl blue tetrazolium bromide (MTT), phosphate buffered saline (PBS), fetal bovine serum (FBS), penicillin–streptomycin solution and McCoy’s medium, dimethyl sulphoxide (DMSO), low melting point (LMP) agarose, normal melting point (NMP) agarose, ethylenediaminetetraacetic acid (EDTA), triton X-100, Sybr Green, iron(III) chloride (FeCl_3_), iron(II) sulfate heptahydrate (FeSO_4_·7H_2_O), α-amylase, α-glucosidase, 3,5-dinitrosalicylic acid (DNS), *p*-nitrophenyl α-D-glucopyranoside (pNPG), 2,4,6-tris(2-pyridyl)-*S*-triazine (TPTZ), Trizma base, sodium potassium tartrate, potassium phosphate monobasic (KH_2_PO_4_), potassium phosphate dibasic (K_2_HPO_4_), 2,4-dinitrobenzene chloride (CDNB), 2,6-dichlorophenolindophenol sodium salt hydrate (DCPIP), β-nicotinamide adenine dinucleotide phosphate (NADPH), glutathione (GSH) from Sigma-Aldrich (Steninheim, Germany). Standard compounds were purchased from quercetin, 6-hydroxy-2,5,7,8-tetramethylchroman-2-carboxylic acid (Trolox) and cyanidin-3-*O*-glucoside were purchased from Sigma Aldrich (Steinheim, Germany). HPLC grade and analytical grade methanol were purchased from Chempur (Piekary Śląskie, Poland), analytical grade ethyl acetate was obtained from POCH (Gliwice, Poland). Formic acid and Folin–Ciocalteu’s phenol reagent (FC) were from Merck (Darmstadt, Germany). OxiSelect™ Cellular Antioxidant Activity Assay Kit was from CellBioLabs (San Diego, CA, USA). Water was purified with a QPLUS185 system from Millipore (Bedford, MA, USA). 

### 2.2. Plant Material

Yellow and red raspberries (*Rubus idaeus* L.), white and red grapes (*Vitis vinifera* L.), white (*Morus alba* L.) and black (*Morus nigra* L.) mulberries, and white (*Ribes rubrum* L.), red (*Ribes rubrum* L.), and black (*Ribes nigrum* L.) currants were purchased from a local market in the Northern Poland. Fruits were stored under freezing conditions between purchase and sample preparation. Samples (about 200 g) were lyophilized (Alpha 2-4 Christ LDC, Osterode, Germany), pulverized, and stored at −20 °C until extract preparation. 

### 2.3. Plant Extract Preparation

Extracts were prepared according to the following procedure: fruit lyophilizates were suspended in deionized water (0.06 g/mL) and the suspensions were treated with ultrasound for 15 min. Then, in order to remove particulates, samples were centrifuged (Heraeus Megafuge 16R Centrifuge, Thermo Fisher Scientific, Osterode, Germany) at 2800× *g* for 20 min at 4 °C and sterilized by passing through Millex sterile R33 mm (0.22 μm) syringe-driven filters (Millipore, Bedford, MA, USA). Water was chosen for extraction because in biological tests this solvent showed the lowest influence on test results, ethanol and methanol exhibit significant toxicity towards tested cells. Additionally, water is more accurate as it is a natural component of fruits and vegetables, and most of the enzymatic reactions occur in the aqueous environment.

### 2.4. Cell Culture

Human colon adenocarcinoma cells (HT29; ATCC cell culture collection, Monassas, VA, USA) were grown in Smart cell incubator (Heal Force; 37 °C, 5% CO_2_). McCoy’s medium supplemented with L-glutamine (2 mol/L), sodium pyruvate (200 g/L), fetal bovine serum (100 mL/L), and antibiotics (10,000 U/mL penicillin and 100 g/L streptomycin) were used as suggested by ATCC. Cultured cells were regularly checked for mycoplasma contamination by the use of universal Mycoplasma Detection Kit (ATCC, Monassas, VA USA). Cells were used for the determination of various biological activities of tested berry fruit extracts. 

### 2.5. Composition of Phenolic Compounds

Phenolic compounds were analyzed according to the procedure described by Kusznierewicz et al. [21] using the Agilent 1200 Series HPLC-DAD-MS system (Agilent Technologies, Santa Clara, CA, USA) with Phenomenex Kinetex XB-C18 column (150 × 4.6 mm, 3.5 μm). The separation conditions were as follows: mobile phase: A: 4.8% (*v*/*v*) formic acid in water, B: methanol; elution program: 0 min—2% B, 30 min—50% B; flow rate: 0.8 mL/min, injection volume: 10 μL; column temperature: 21 °C. If standard compounds were not available, the basis for the identification of individual components were a comparison of retention time, mass signal [M+H]^+^, [M+H]^−^ and fragment ions (*m/z*) with available literature data. The content of phenolic compounds in fruit extracts was determined using the external standard method. To determine the concentration of individual analytes, the equation of the calibration curve prepared for the appropriate standard or the main representative of the group of phenolic substances was used: chlorogenic acid for derivatives of hydroxycinnamic acid (325 nm), gallic acid for derivatives of hydroxycinnamic acid (270 nm), quercetin for flavonols (360 nm), cyanidine glucoside for anthocyanins, and catechin for flavan-3-ols (270 nm).

### 2.6. Profiles of Antioxidants and Antioxidant Capacity

Profiles of antioxidants present in studied berry fruits were obtained by HPLC-DAD (Agilent Technologies, Santa Clara, CA, USA) coupled with post-column derivatization with ABTS reagent using a Pinnacle PCX Derivatization Instrument (Pickering Laboratories, Inc., Mountain View, CA, USA) according to the procedure described previously [21,22]. 

Standard spectrophotometric methods (ABTS, DPPH, and FC) were used to determine antioxidant activity of tested fruit extracts as described earlier [22,23]. Obtained results were expressed as Trolox Equivalents (TE (μmol/g d.w.)).

FRAP assay performed using the method described previously by Benzie and Strain [24] was used to determine reducing potential (FeSO_4_·7H_2_O mg/g dry weight (d.w.)) of tested fruit extracts. 

OxiSelect Cellular Antioxidant Activity Assay Kit (Cell Biolabs Inc. Cat. No. STA-349, San Diego, CA, USA) was used to obtain cellular antioxidant activity of tested fruit extracts. The treatment of the cells was conducted as described previously [20]. The procedure applied to measure the antioxidant activity followed strictly producer’s recommendations. Results were expressed as Quercetin Equivalents (QE (μmol/g d.w.)). 

### 2.7. Cytotoxic Activity towards HT29 Cells

The cytotoxic effect of studied fruit extracts on human colon cancer (HT29) cells was determined using MTT assay protocol described previously [25] and expressed as percentage of growth of untreated cells (% control) regarded as 100%. 

### 2.8. Antigenotoxic Activity

The ability of tested fruit extracts to protect DNA from oxidative damage was assessed using the comet assay procedure described previously [26]. Briefly, HT29 cells after they reached about 80% confluence were treated with fruit extracts (final concentration 10% *v*/*v*) for 24 h. Next they were treated for 1 h with H_2_O_2_ (final concentration 0.1 mM) that served as a model oxidative DNA damage inducer. The positive control was cells treated only with 0.1 mM H_2_O_2_, and as negative control—nontreated cells. After cell treatment, a comet assay procedure was applied as described elsewhere [26]. DNA comets were examined under fluorescent microscope (Carl Zeiss AxioImager.Z2) connected to a scanning system Metafer 4 (MetaSystem, Zeiss). Then, 200 consecutive comets were counted for each slide. % DNA in Tail parameter was used to express DNA damage.

### 2.9. Influence on Activity of Phase II Detoxification Enzymes

#### 2.9.1. Preparation of Cytosolic Fractions

After HT29 cells reached roughly 80% confluence, they were treated with tested fruit extracts (final concentration in the medium 10% *v*/*v*) for 6 or 24 h at 37 °C. Procedure used for cytosolic fraction preparation was described previously [20]. The cytosolic protein content was determined using Bicinchoninic Acid Protein Assay Kit (Sigma Aldrich, Cat. No. BCA1 AND B9643, Saint Louis, MO, USA). Prepared cytosolic fractions were used for the determination of the influence of tested fruit extracts on the activity of phase II detoxification enzymes (Glutathione S-Transferase (GST) and Quinone Oxidoreductase (NQO1)).

#### 2.9.2. GST Activity

GST activity was determined using Habig’s method with slight modifications, described previously [27]. Briefly, 0.94 mL of CDNB (1 mmol/L) was mixed with 0.05 mL of glutathione (GSH, 20 mmol/L) in a spectrophotometric cuvette. The absorbance measurement was carried out for 3 min at 340 nm at 37 °C (Nanodrop 2000c, Thermo Scientific, Valtham, MA, USA) in order to determine the background reaction rate. Next, 0.01 mL of cytosolic fraction was added, and absorbance was measured for another 10 min to determine the rate of reaction catalyzed by GSTs. The GST activity was expressed as nmol CDNB/ min·mg protein.

#### 2.9.3. NQO1 Activity

The NQO1 activity was determined using the method described previously [27]. Briefly, the 0.1 mL of KCl-phosphate buffer (0.1 mol/L, pH 7.7) was mixed with 0.03 mL of 1.2 mmol/L DCPIP solution, and 0.8 mL of water in spectrophotometric cuvette. The absorbance of this mixture (background) was measured at 600 nm at 37 °C for 3 min (Nanodrop 2000c, Thermo Scientific, Valtham, MA, USA). Then, 0.03 mL of freshly prepared NADPH (6 mmol/L) and 0.03 mL of previously prepared cytosolic fraction were added to the cuvette and measurements continued for another 10 min. Control samples contained 0.03 mL of buffer instead of cytosolic fraction. The NQO1 activity was expressed as nmol DCPIP/ min·mg protein. 

### 2.10. Antidiabetic Activity

#### 2.10.1. Inhibition of α-Amylase Activity

For the determination of the influence of studied extracts on α-amylase activity the protocol described previously by Zia-Ul-Haq et al. [28] was used. Briefly, 20 μL of plant extract or Cy-3-*O*-Glu solution was mixed with 20 μL of a-amylase (0.05 U/μL) in 0.1 M phosphate buffer (pH 6.9). Samples were incubated for 5 min at 37 ° C. In parallel, the control sample was prepared, to which 20 μL of 0.1 M phosphate buffer (pH 6.9) was added instead of the plant extract or Cy-3-*O*-Glu solution. In addition, background samples for each sample and control were prepared, to which 20 μL of phosphate buffer (pH 6.9) was added instead of the enzyme. Each sample was prepared in three independent replicates. After the incubation, 250 μL starch solution (1% *w*/*v*) was added to each sample (also to control and backgrounds). Samples were mixed and then incubated for 10 min at 37 °C. After incubation, 200 μL DNS solution (1% *w*/*v*) was added to all samples. The samples were incubated for 15 min at 100 °C to stop the enzymatic reaction. The samples were cooled to room temperature and diluted prior to measurement by adding 5 mL of distilled water. The absorbance of the solutions was measured at λ = 540 nm. The results were expressed as % inhibition of α-amylase activity by studied fruit extracts or Cy-3-*O*-Glu solution compared to control noninhibited reaction.

#### 2.10.2. Inhibition of α-Glucosidase Activity

For the determination of the influence of tested plant extracts on α-glucosidase activity the procedure described earlier by Zia-Ul-Haq et al. [28] was used. Briefly, 100 μL of the tested plant extract or Cy-3-*O*-Glu solution was mixed with 200 μL of α-glucosidase solution (1 U/mL) in phosphate buffer (0.1 M, pH 6.9). The samples were incubated for 10 min at 37 °C. In parallel, control sample was prepared, in which 100 μL of 0.1 M phosphate buffer (pH 6.9) was added instead of the plant extract. In addition, background samples were prepared with 0.1 M phosphate buffer instead of the enzyme. After the incubation period, 600 μL phosphate buffer (0.1 M, pH 6.9) was added to all samples. Next 100 μL of pNPG solution (5 mM, in phosphate buffer pH 6.9) was added. The reaction was carried out for 5 min at 37 °C. The enzyme reaction was stopped by adding 50 μL sodium carbonate (2 M). The absorbance was measured at λ = 405 nm. The results were expressed as % inhibition of α-glucosidase activity by studied fruit extracts or Cy-3-*O*-Glu solution compared to control noninhibited reaction.

### 2.11. Statistical Analysis

Unless stated otherwise, all values represent means ± SD (standard deviation) of three independent experiments. Correlations between food matrix composition, antioxidant activity, the contents of phenolic compounds, and biological activity of plant samples were examined using Pearson’s coefficients. Statistical differences among samples were tested using one-way analysis of variance (ANOVA) with Dunnets’ or Tukey post hoc test as appropriate (GraphPad Prism 4). The correlations between tested activities and types of extracts were determined by PCA analysis (TurboPascal).

## 3. Results

### 3.1. Composition of Phenolic Compounds 

The composition of polyphenols present in studied extracts from yellow (YRas) and red (RRas) raspberries, white (WGra) and red (RGra) grapes, white (WMul) and black (BMul) mulberries, and white (WCur), red (RCur), and black (BCur) currants was determined by high-performance liquid chromatography with diode-array and mass spectrometry detectors (HPLC-DAD-ESI-MS). The content of identified polyphenols is given in Table 1; the full list of detected redox-active components is presented in the Supplementary Materials (Table S1). Analysis of obtained data confirmed that pigmented varieties of tested fruits are richer sources of antioxidants than their colorless counterparts (Table 1). 

One of the most important groups of phenolic compounds with well documented health-promoting properties present in studied pigmented fruits are anthocyanins. BMul occurred to be the richest source of these compounds (11.708 mg/g d.w.), followed by RRas (3.142 mg/g d.w.), BCur (1.631 mg/g d.w.), RGra (0.962 mg/g d.w.), and the poorest source among pigmented varieties was RCur (0.425 mg/g d.w.) (Table 1). Although the content of anthocyanins was the highest in BMul, RGra extract was characterized by the most diverse profile of anthocyanins among all tested fruits (14 different compounds, while BMul only had four). The obtained results are in accordance with those reported in [29], where the same six anthocyanins in BCur extract were identified. Määttä et al. [10] and Rubinskine et al. [30] identified the same four main anthocyanins in BCur extract. Corresponding three main anthocyanins in BCur extract (11 total), two in RCur extract, and eight in RRas extract were identified by Borges et al. [31]. Colorless varieties of studied fruits did not contain anthocyanins.

An important group of phenolic compounds present in significant amounts in tested fruits were derivatives of hydroxycinnamic and hydroxybenzoic acids. The richest source of these compounds was RRas (6.01 and 1.08 mg/g d.w., respectively), followed by BMul (1.74 and 2.87 mg/g d.w.) and YRas (1.98 and 0.98 mg/g d.w.). Phenolic acids were the only phenolic compounds present in YRas extract (Table 1) and the most abundant group detected in RRas, WCur, RCur, and WGra. 

Another group of polyphenols—flavan-3-ols (mostly catechin and epicatechin)—were found in RRas (0.74 mg/g d.w.), RGra (2.01 mg/g d.w.), WMul (1.89 mg/g d.w.), and BMul (8.47 mg/g d.w.). These flavonoids were not detected in extracts from other tested fruits (Table 1). 

**Figure 1:**
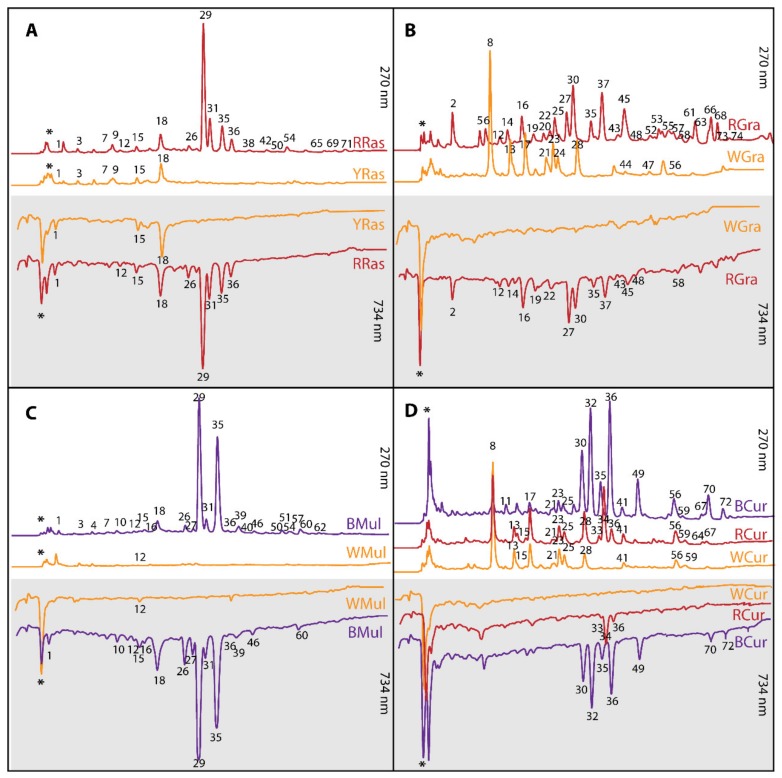
Sample high-performance diode array detector (HPLC-DAD) chromatograms of water extracts from (**A**) yellow (YRas) and red (RRas) raspberries; (**B**) white (WGra) and red (RGra) grapes; (**C**) white (WMul) and black (BMul) mulberries; (**D**) white (WCur), red (RCur), and black (BCur) currants. Chromatograms traced at 270 nm (white background) are combined with profiles of antioxidants detected online with ABTS (2,2-azinobis-(ethyl-2,3-dihydrobenzothiazoline-6-sulphonic acid) diammonium salt) radical monitored at 734 nm (grey background). The numbers of peaks correspond to analytes listed in Table 1 and Table S1 (Supplementary Materials); * unidentified polar compounds.

The last group of phenolic compounds identified in studied fruits were flavonols, and among them quercetin and rutin were the most abundant representatives. These compounds were detected in all tested fruits besides YRas and WMul (Table 1). The content of flavonols was very low in comparison to other phenolic compounds and they did not show a significant influence on the antioxidant capacity of tested fruit extracts (Figure 1). BMul was again the richest source of these phytochemicals (1.49 mg/g d.w.).

### 3.2. Antioxidant Capacity and Profiles of Antioxidants

Profiles of antioxidants detected in tested extracts with the aid HPLC coupled with post-column derivatization with ABTS radical are shown in Figure 1. Such profiles not only reveal the individual analytes with antioxidant potential, but also enable quantitation of their input into the antioxidant potential of tested samples. The obtained antioxidant profiles revealed that in the case of pigmented varieties the main individual contributors to the antioxidant activity were anthocyanins, while in the colorless varieties, these were mostly unidentified polar compounds (marked on profiles as *) embracing ascorbic acid among others. According to these results, among all tested samples, the pigmented varieties were characterized by richer antioxidant profile and stronger antioxidant potential than their colorless counterparts. The richest profile of antioxidants was revealed for RGra, followed by BCur, BMul, and RRas. 

**Figure 2:**
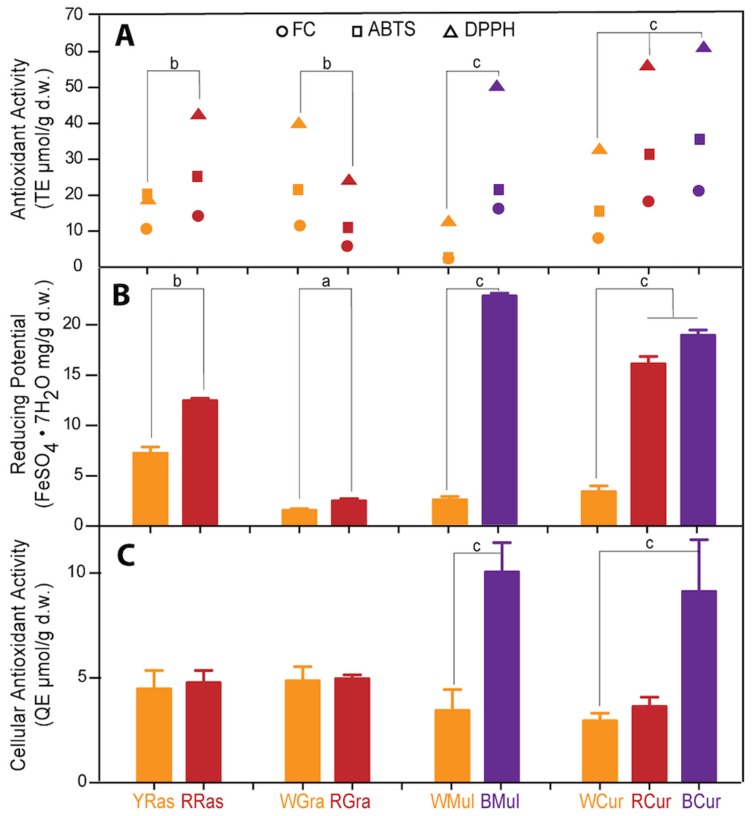
Antioxidant capacity assessed for water extracts from yellow (YRas) and red (RRas) raspberry, white (WGra) and red (RGra) grapes, white (WMul) and black (BMul) mulberry, and white (WCur), red (RCur), and black (BCur) currant varieties. (**A**) Antioxidant capacity determined by standard spectrophotometric tests FC, DPPH, and ABTS (expressed as Trolox equivalents; TE μmol/g d.w.). (**B**) Reducing potential determined by FRAP assay (expressed as ferric ion reducing antioxidant parameter; FeSO_4_·7H_2_O mg/g d.w.). (**C**) Cellular antioxidant activity assessed by CAA test in human colon adenocarcinoma (HT29) cells (expressed as quercetin equivalents; QE μmol/g d.w.). The results represent means ± SD of three independent determinations. Significantly different values determined by one-way analysis of variance (ANOVA) with Tukey post hoc test are marked as ^a^ *p* < 0.05, ^b^ *p* < 0.01, ^c^ *p* < 0.001.

Antioxidant capacity of tested plant extracts was assessed in two ways: in a cell-free system using standard chemical tests (FC, DPPH, ABTS, and FRAP) and in human colon cancer HT29 cells by CAA assay (Cellular Antioxidant Activity assay). The determined antioxidant capacities of tested plant extracts obtained using spectrophotometric batch tests (FC, DPPH and ABTS) are assembled in Figure 2, and were highly correlated (Pearsons’ coefficients: FC vs. DPPH—0.934; FC vs. ABTS—0.976; ABTS vs. DPPH—0.889). With the exception of grapes, for all pairs of fruits, pigmented varieties showed stronger antioxidant capacity than the colorless ones due to the presence of anthocyanin fraction, as clearly seen in Figure 1. In these batch tests, BCur extract exhibited the strongest antioxidant capacity of all tested samples, followed by RCur > BMul > RRas > WGra > RGra > YRas > WMul. The results obtained using FRAP assay (Figure 2) were correlated with other batch tests (FRAP vs. FC—0.823; FRAP vs. DPPH—0.784; FRAP vs. ABTS—0.703) and fail to match only the ones obtained by other chemical tests only for grapes.

In the FRAP assay, all pigmented varieties of fruits showed stronger reducing potential than their colorless counterparts. The strongest reducing potential was displayed by BMul extract, followed by BCu > RCur > RRas > YRas > WCur > WMul > RGra > WGra. 

Finally, cellular antioxidant capacity of tested fruit extracts was assessed by CAA test in HT29 cells that served as a model of human alimentary tract. The drawback of chemical antioxidant activity assays is that they are performed under nonphysiological pH and temperature conditions, and neither take cell metabolism into consideration, so unlike CAA test, so they can hardly be used to predict redox activity under in vivo conditions. These limitations are somewhat overcome in cellular assays, whose results often differ from those obtained by batch tests. As can be seen in Figure 2, only two pigmented varieties showed stronger antioxidant capacity than colorless ones in the CAA test. These two were BMul and BCur, for which differences were statistically significant (Figure 1). Contrary to batch tests, but similarly to FRAP (Pearsons’ coefficients: CAA vs. FRAP—0.748), BMul showed the strongest cellular antioxidant activity.

### 3.3. Chemopreventive Potential of Tested Plant Extracts in Cellular Model

**Figure 3:**
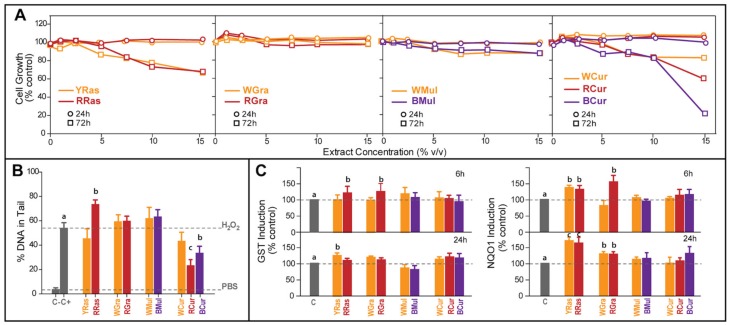
The chemopreventive potential of water extracts from yellow (YRas) and red (RRas) raspberry, white (WGra) and red (RGra) grapes, white (WMul) and black (BMul) mulberry, and white (WCur), red (RCur), and black (BCur) currant varieties. (**A**) Influence on the growth of HT29 cells exposed to fruit extracts studied for two incubation periods (24 and 72 h). The results represent means of three independent determinations (SD did not exceed 5%). (**B**) The ability of tested water fruit extracts to protect DNA from oxidative damage assessed by comet assay. Negative control (C-)—cells not treated with genotoxic reagent; positive control (C+)—cells treated with 100 μM H_2_O_2_. The results represent means ± SD of three independent determinations. Significantly different values determined by one-way analysis of variance (ANOVA) with Dunnett’s post-test are marked as ^b^ *p* < 0.05, ^c^ *p* < 0.01. (**C**) The induction of phase II detoxification enzymes: glutathione transferases (GST) and quinone oxidoreductase (NQO1) in HT29 cells exposed to tested plant extracts (10% *v*/*v* in medium) for 6 h or 24 h. C—nontreated control cells. The results represent means ± standard deviation (SD) of three independent experiments. Significantly different values determined by one-way analysis of variance (ANOVA) with Dunnett’s post hoc test are marked as ^b^ *p* < 0.05, ^c^ *p* < 0.01.

The ability to slow down proliferation of cancer cells is frequently implicated in the assessment of chemopreventive properties of plant extracts containing bioactive phytochemicals. In our study, human colon cancer cells were exposed to different concentrations of tested fruit extracts for 24 and 72 h (Figure 3A). After 24 h of incubation with plant extracts, no impact on cell growth was observed. In the case of prolonged incubation (72 h), raspberry and currant extracts (all varieties) showed growth inhibiting potential (at concentrations above 5% *v*/*v*). In the case of raspberries, both varieties showed similar cytotoxic potential, while for currants the strongest cytotoxic effect was observed for BCur extract followed by RCur and WCur.

Antigenotoxic effect of tested plant samples was evaluated by pretreating HT29 cells for 24 h with 10% (*v*/*v*) of tested berry fruit extracts, and then exposing them for 1 h to H_2_O_2_ (100 μM) used as an oxidative stress inducer. Under test conditions only extracts from RCur and BCur showed statistically significant ability (*p* < 0.05 and *p* < 0.01, respectively) to protect DNA from damage caused by oxidative stress (Figure 3B). RRas enhanced the oxidative damage to DNA (*p* < 0.05). Both varieties of grapes and mulberries did not significantly affect DNA integrity in cells exposed to oxidative stress.

The stimulation of phase II detoxification enzymes is yet another common biomarker of chemopreventive potential. In our study (Figure 3C), only raspberry and grape extracts displayed significant ability (*p* < 0.05) to increase the activity of phase II detoxification enzymes (GST, NQO1) after both tested incubation times (6 and 24 h). The presence of anthocyanins did not cause substantial difference for the induction of these enzymatic activities.

### 3.4. Inhibition of α-Amylase and α-Glucosidase in a Cell-Free Model

The development of type 2 diabetes is associated with the activity of gut enzymes catalyzing the degradation of starch—α-amylase and α-glucosidase. Therefore, their inhibitors are believed to be useful in prevention of early stages of diabetes by modulating the breakdown of carbohydrates. In this study, we investigated whether bioactive phytochemicals present in berry fruit extracts can influence the activity of these enzymes.

**Figure 4:**
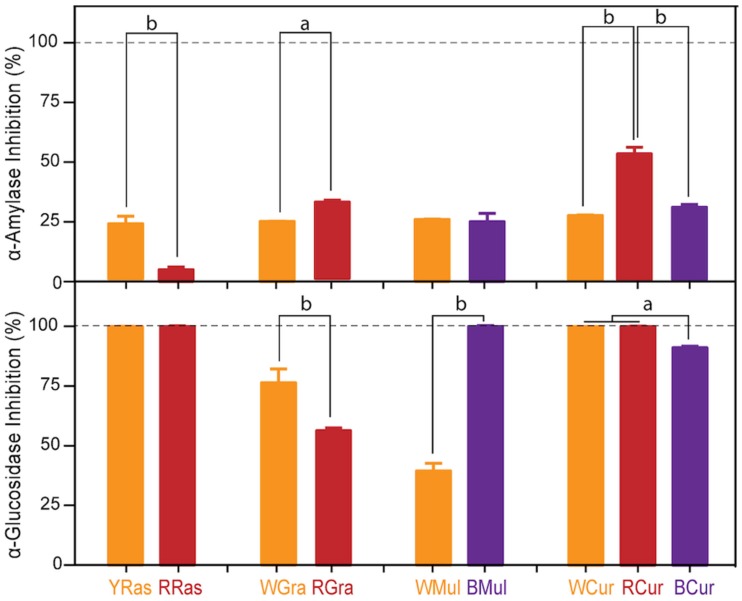
Inhibition of α-amylase and α-glucosidase by water extracts from yellow (YRas) and red (RRas) raspberries, white (WGra) and red (RGra) grapes, white (WMul) and black (BMul) mulberries, and white (WCur), red (RCur), and black (BCur) currants. The results represent means ± SD of three independent determinations. The results represent means ± SD of three independent experiments. Significantly different values determined by one-way analysis of variance (ANOVA) with Tukey’s post hoc test are marked as ^a^ *p* < 0.05, ^b^ *p* < 0.01.

All studied extracts affected the activity of both enzymes to various degrees. These inhibitory properties differed significantly between majority of colored vs. colorless varieties (*p* < 0.05, *p* < 0.01), but there was no trend related to anthocyanin content. In the case of α-amylase (Figure 4), the strongest inhibition of this enzyme was observed for RCur (53%), followed by RGra (32%), and BCur (30%). The strongest inhibition of α-glucosidase activity was observed for YRas, RRas, BMul, WCur, and RCur (100% inhibition), followed by BCur (91%). 

### 3.5. Chemopreventive Properties of Cy-3-O-Glu

**Figure 5:**
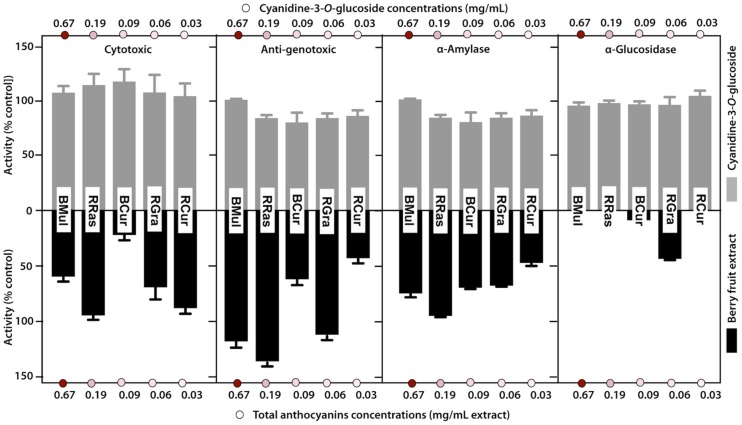
Comparison of biological activities of crude berry fruit extracts (bottom graphs) and solutions of purified cyanidine-3-*O*-glucoside (Cy-3-*O*-Glu) (top graphs) used at concentrations corresponding to the total anthocyanin content in corresponding extracts. Panel 1: cytotoxic activity of tested samples towards human colon cancer cells (HT29) after 72 h incubation with samples at concentration 15% (*v*/*v*); Panel 2: antigenotoxic activity of tested samples; Panel 3: influence of tested samples on α-amylase activity. Panel 4: influence of tested samples on α-glucosidase activity. All data represent means ± SD of three independent determinations.

The results obtained in this study suggested that anthocyanins do not have a significant influence on tested biological activities with the exception of antioxidant activity, however, even in this case the outcome was not uniform. To verify the initial presumption that the content of pigment may impact biological properties of colored fruits, the influence of purified cyanidin-3-*O*-glucoside (Cy-3-*O*-Glu) solution on investigated biological activities (cytotoxic, antigenotoxic activity, and the ability to influence α-amylase and α-glucosidase activity) was studied. The applied concentrations of Cy-3-*O*-Glu solution reflected the total concentration of anthocyanins in appropriate colored plant samples. The obtained data on biological activities were compared with those obtained for crude plant extracts (Figure 5). 

The solution of purified Cy-3-*O*-Glu at concentrations corresponding to the total anthocyanin content determined for studied fruit extracts, did not show a cytotoxic effect in HT29 cells over the whole range of treatments (Figure 5). The results obtained in the comet assay suggested that the observed protection of DNA against oxidative damage did not result from the presence of anthocyanins in tested fruits, otherwise all pigmented varieties should elicit a dose-dependent DNA protecting effect. This observation was confirmed in experiments with purified Cy-3-*O*-Glu, that also did not show any DNA protective effect (exceeding 100% control), in contrast to some colored fruit extracts (RCur, BCur) (Figure 5). 

Moreover, opposite to crude plant extracts that proved to be effective inhibitors of antidiabetic enzymes, purified Cy-3-*O*-Glu did not statistically significantly inhibit α-amylase or α-glucosidase (Figure 5). 

## 4. Discussion

Chromatographic analysis of tested fruit extracts showed that colored varieties were richer sources of antioxidants than colorless ones, and that the latter were devoid of pigments from the anthocyanin group. 

Further chemical characterization of extracts was in the form of antioxidant profiling by HPLC coupled with post-column derivatization with ABTS radical, which showed antioxidant activity of individual compounds. In turn, batch spectrophotometric tests were performed for mixtures as a whole, so antioxidant capacity was influenced by interactions between redox active components as well as plant matrix. We expected that colored varieties, because of the presence of anthocyanins (which belong to very strong plant antioxidants), would exhibit higher antioxidant capacity. This hypothesis was proved by previous studies of Sumaya-Martinez [15], Sanchez-Sulcedo et al. [16], Matsafiji et al. [13], Kim et al. [18], Garcia-Lafuente et al. [19], Koss-Mikołajczyk et al. [9], and Koss-Mikołajczyk et al. [20] that showed that antiradical activity was significantly correlated with the concentration of redox active compounds. This turned out not to be the case for RGra in batch spectrophotometric tests (Figure 2), although antioxidant profiles revealed substantial antioxidant activity of RGra pigments. Apparently, the interactions between different redox active components influenced the total antioxidant capacity of the extracts in batch tests. When one compares antioxidant activities (Figure 2) with chemical composition of extracts (Table 1) certain interactions are especially visible. The presence of catechins seems to diminish the antioxidant input of anthocyanins. RRas containing both catechins and anthocyanins displayed lower antioxidant capacity than BCur devoid of catechins, although it contains twice as many anthocyanins. BMul, which is the richest source of catechins and cyanidins, showed only little stronger antioxidant activity than BCur lacking catechins and containing seven times less anthocyanins. 

In contrast to all other tests, FRAP assay results suggested that reducing potential in this case correlated best with flavonol + hydroxycinnamates content. The latter polyphenols were particularly abundant in raspberries, which showed surprisingly high reducing potential in this test compared to other berry fruit extracts studied. CAA results were at a similar level with the two samples standing out, i.e., extracts from BMul and BCur that contained the highest concentration of flavonoids. Thus, as already mentioned, cellular effectiveness of exogenous redox-active compounds cannot be reliably predicted based on chemical antioxidant activity tests. 

In order to find the possible correlation between phenolic compounds composition and biological activities, apart from antioxidant activity, we assessed some other chemopreventive properties, that have been previously reported for anthocyanins, berry fruit extracts studied, or purified Cy-3-*O*-Glu. In our previous study [20], we observed a strong correlation between the content of phytochemicals and antioxidant capacity of tested extracts, but no such straightforward relationship between the chemical composition and chemopreventive potential, while comparing betalain content and composition with biological activities (cytotoxicity, protection against reactive oxygen species (ROS), and influence on selected enzymatic activities) of extracts from white and red beetroot and yellow, orange, and red prickly pear varieties. Similarly, a study by our group compared the health promoting potential and composition of bioactive phytochemicals for differently pigmented cabbage (white and red) and cauliflower (white and purple) varieties and we showed that again no simple relationship between these parameters can be indicated [9]. Other studies also did not show a direct correlation between content of bioactive phytochemicals and biological activities of tested plants. For instance, Shon et al. [17], besides the content of phenolic compounds in white, yellow, and red onion, compared also their antioxidant capacity and antimutagenic potential. This study showed a very weak relationship between the content of bioactive phytochemicals and biological activities displayed by extracts from tested onion varieties. All tested extracts displayed similar antimutagenic activity. Kim et al. [18] in their study on white and red pitaya also did not observe any correlation between phenolic content and antiproliferative activity, though they showed a strong correlation between the phenolic content and antioxidant effect.

It has been documented in a number of studies that phenolic compounds present in berry fruits are responsible for both cell cycle arrest and cancer cell growth inhibition. Jia et al. [29] confirmed that the high concentration of phenolic compounds present in black currants is responsible for their antiradical activity and anticarcinogenic effects. Morphological observations with inverted fluorescence microscope clearly revealed cell shrinkage, formation of cytoplasmic filaments, nuclear chromatin condensation, and cell apoptosis in cancer cells treated with black currant extract. Black currant extract has also suppressed the growth of human colon (HT29, HCT-116), breast (MCF-7), and leukaemia (HL-60) cancer cells [32,33,34]. These results suggest that anthocyanins were not responsible for the observed cytotoxic effects, because pigmented varieties of tested fruits (except for currants) did not show a stronger ability to inhibit cell growth than colorless ones. Although plant extracts differed in their cytotoxic activity (for 72 h incubation), Cy-3-*O*-Glu at the same concentration as in studied extracts did not show any cytotoxicity, thus cell growth inhibition must have resulted from the exposure to other plant components or more likely their combinations. Our results are in line with the findings of Bishayee et al. [35], who showed that anthocyanin-rich fraction of BCur extract exhibited a potent cytotoxic effect in HepG2 cells and this effect was more pronounced than that of delphinidin and cyanidin, two major aglycones of anthocyanins present in BCur. These results indicate that there might possibly be some additive or synergistic effects between components of BCur skin extracts. Additionally, results of our previous study on differently pigmented cabbage and cauliflower varieties suggested that their ability to inhibit growth of cancer cells was modulated not only by polyphenols and glucosinolates present in studied plants, but also by other components of these vegetables [9]. 

Another biological activity that is often used to evaluate the chemopreventive potential of bioactive phytochemicals is their ability to protect DNA from damage induced by oxidative stress [9,20]. The results obtained by comet assay showed that the ability to prevent oxidative DNA damage was not correlated with the content of pigments. In some cases, colored varieties indeed showed protective effect (currants), however, others caused significant enhancement of DNA fragmentation to occur (RRas) while for grapes and mulberry no difference between varieties was seen under the conditions of the experiment. Similarly, as in the case of cell growth inhibition, the differences between plant extracts detected by comet assay were not matched by corresponding treatments with purified Cy-3-*O*-Glu solutions. Such conclusions were also drawn in our previous study on differently pigmented cabbage and cauliflower varieties, where no correlation was found between anthocyanin content and DNA protecting effect of differently pigmented cabbage and cauliflower extracts [9].

Evidence has also accumulated that anthocyanins and other phenolic compounds present in berry fruits are able to stimulate phase II detoxification enzymes’ activity [36,37]. This is another property used as a biomarker of chemopreventive potential. In our study, activity of both studied phase II detoxification enzymes (GST, NQO1) was not influenced in a differentiated way by pigmented vs. colorless varieties of tested fruits. Such an activity was detected for raspberries and grapes, but regardless of their color. Our previous study also showed that anthocyanin content did not influence activity of phase II detoxification enzymes in cabbage or cauliflower [9]. 

Studies with cell lines, animal models, and clinical trials provide some evidence that anthocyanins may be useful in control of diabetes. Published data support the hypothesis that anthocyanins lower blood glucose as a result of overcoming insulin resistance, protection of β cells, stimulated secretion of insulin, and slowing down digestion of sugars in the small intestine. These mechanisms are mostly related to antioxidant properties of these polyphenols, but inhibition of digestive enzymes and other pathways cannot be excluded [38]. This study failed to answer the question regarding which compound(s) present in studied berries were behind the observed inhibition of α-amylase and α-glucosidase, because no dose-dependent response could be related to bioactive phytochemical composition of tested extracts. All tested plant extracts turned out to be good inhibitors of both enzymes, while in the case of purified Cy-3-*O*-Glu solutions no enzyme inhibition was observed. In contrast to our results, McDougal et al. [39] showed that the extent of inhibition of α-glucosidase by tested soft-fruit extracts was correlated with their anthocyanin content. For instance, blueberry and blackcurrant extracts, with the highest anthocyanin content, were the most effective inhibitors of α-glucosidase. On the other hand, strawberry and raspberry extracts, that contained appreciable amounts of soluble tannins, were most effective in inhibiting α-amylase. 

## 5. Conclusions

In summary, no clear cut relationship between the content and composition of bioactive phytochemicals and chosen biological activities exhibited by studied berry fruit extracts was observed. The richest source of phenolic compounds (phenolic acids, flavonols, flavan-3-ols, and anthocyanins) turned out to be black mulberry (BMul), followed by red raspberry (RRas), and black currant (BCur). The only activity that was unambiguously related to anthocyanins content was antioxidant capacity as confirmed PCA analysis; the results of antioxidant activity tests accounted for 49% of variation. None of other tested biological properties correlated with the content of anthocyanins. Moreover, the experiments with the use of purified anthocyanin (Cy-3-*O*-Glu) solutions at concentrations corresponding with the total anthocyanin contents in studied fruit extracts confirmed that anthocyanins were not significantly influencing biological activity of tested fruit extracts. One can thus presume that there must be some type of interaction between different groups of bioactive phytochemicals involved in the health-promoting activity of the tested plant extracts as suggested by e.g., the food synergy concept. 

## Figures and Tables

**Table 1:** Polyphenolic compound composition of berry fruit extracts studied.

^a^ Peak No	Compounds (mg/g d.w.)	YRas	RRas	WGra	RGra	WMul	BMul	WCur	RCur	BCur
1–4, 6–10, 13, 18, 20, 22, 25, 28, 41–42, 54, 62, 65, 69, 71	Σ Hydroxybenzoic acid derivatives	0.976 ± 0.033	1.078 ± 0.153	0.911 ± 0.035	0.346 ± 0.083	0	2.872 ± 0.223	0.895 ± 0.023	0.968 ± 0.048	0.478 ± 0.064
5, 11, 14–15, 17, 21, 23–24, 26	Σ Hydroxycinnamic acid derivatives	1.978 ± 0.083	6.009 ± 0.646	0.861 ± 0.022	0.203 ± 0.040	0	1.744 ± 0.157	0.465 ± 0.101	0.791 ± 0.085	0.650 ± 0.077
12, 16, 19, 27	Flavan-3-ols									
12	Catechin	nd	0.738 ± 0.045	nd	0.278 ± 0.074	0.177 ± 0.005	1.888 ± 0.161	nd	nd	nd
27	Epicatechin	nd	nd	nd	0.642 ± 0.096	nd	2.836 ± 0.282	nd	nd	nd
	Σ Flavan-3-ols	0	0.738 ± 0.045	0	2.008 ± 0.085	1.888 ± 0.161	8.465 ± 0.826	0	0	0
38, 40, 44, 47–51, 55–57, 59–60, 64, 67	Flavonols									
60	Quercetin	nd	nd	nd	nd	nd	0.195 ± 0.003	nd	nd	nd
56	Rutin	nd	nd	0.134 ± 0.006	nd	nd	0.201 ± 0.005	0.111 ± 0.007	0.180 ± 0.024	0.065 ± 0.006
	Σ Flavonols	0	0.193 ± 0.015	0.274 ± 0.010	0.259 ± 0.009	0	1.487 ± 0.156	0.181 ± 0.005	0.359 ± 0.031	0.833 ± 0.132
	Anthocyanins									
29	Cy-3-*O*-Soph	nd	1.970 ± 0.045	nd	nd	nd	5.686 ± 0.609	nd	nd	nd
30	Delph-3-*O*-Glu	nd	nd	nd	0.216 ± 0.016	nd	nd	nd	nd	0.315 ± 0.049
31	Cy-3-*O*-(Glu-Rut)	nd	0.507 ± 0.021	nd	nd	nd	0.521 ± 0.063	nd	nd	nd
32	Delph-3-*O*-Rut	nd	nd	nd	nd	nd	nd	nd	nd	0.569 ± 0.068
33	Cy-3-*O*-Samb	nd	nd	nd	nd	nd	nd	nd	0.035 ± 0.001	nd
34	Cy-3-*O*-(Xyl-Rut)	nd	nd	nd	nd	nd	nd	nd	0.314 ± 0.018	nd
35	Cy-3-*O*-Glu	nd	0.458 ± 0.005	nd	0.073 ± 0.003	nd	4.636 ± 0.586	nd	nd	0.161 ± 0.024
36	Cy-3-*O*-Rut	nd	0.206 ± 0.003	nd	nd	nd	0.111 ± 0.018	nd	0.077 ± 0.004	0.483 ± 0.086
37	Pet-3-*O*-Glu	nd	nd	nd	0.205 ± 0.012	nd	nd	nd	nd	nd
39	Pel-3-*O-*Hex	nd	nd	nd	nd	nd	0.381 ± 0.012	nd	nd	nd
43	Peo-3-*O*-Glu	nd	nd	nd	0.011 ± 0.001	nd	nd	nd	nd	nd
45	Malv-3-*O*-Glu	nd	nd	nd	0.142 ± 0.007	nd	nd	nd	nd	nd
46	Cy-3-*O*-(Mal-diGlu)	nd	nd	nd	nd	nd	0.111 ± 0.002	nd	nd	nd
52	Delph-3-*O*-(6-*O*-acetyl)-Glu	nd	nd	nd	0.033 ± 0.003	nd	nd	nd	nd	nd
53	Delph-3-*O*-(6-*O*-p-Coum)-5-diGlu	nd	nd	nd	0.035 ± 0.002	nd	nd	nd	nd	nd
58	Pet-3-*O*-(6-*O-*p-Coum)-5-diGlu	nd	nd	nd	0.046 ± 0.003	nd	nd	nd	nd	nd
61	Pet-3-*O*-(6-*O*-acetyl)-Glu	nd	nd	nd	0.014 ± 0.001	nd	nd	nd	nd	nd
63	Malv-3-*O*-(6-*O*-p-Coum)-5-diGlu	nd	nd	nd	0.025 ± 0.001	nd	nd	nd	nd	nd
66	Malv-3-*O*-(6-*O*-acetyl)-Glu	nd	nd	nd	0.061 ± 0.007	nd	nd	nd	nd	nd
68	Delph-3-*O*-(6-*O*-p-Coum)-Glu	nd	nd	nd	0.015 ± 0.002	nd	nd	nd	nd	nd
70	Peo-3-*O-*Rut	nd	nd	nd	nd	nd	nd	nd	nd	0.102 ± 0.003
73	Pet- 3-*O*-(6-*O*-p-Coum)-Glu	nd	nd	nd	0.056 ± 0.003	nd	nd	nd	nd	nd
74	Malv-3-*O*-(6-*O*-p-Coum)-Glu	nd	nd	nd	0.031 ± 0.002	nd	nd	nd	nd	nd
	Σ Anthocyanins	0	3.142 ± 0.074	0	0.962 ± 0.064	0	11.708 ± 1.329	0	0.425 ± 0.023	1.631 ± 0.105

All values represent means ± SD from three independent measurements. ^a^ Peak No correspond to those in Figure 1. Abbreviations used: Cy—cyjanidin, Delph—delphinidin, Pet—petunidin, Peo—peonidin, Malv—malvidin, Samb—sambubioside, xyl—xylozyl, Coum—coumaryl, Rut—rutinoside, Glu—glucoside, Soph—sophoroside, nd—not detected, d.w.—dry weight, YRas—yellow raspberry, RRas—red raspberry, WGra—white grapes, RGra—red grapes, WMul—white mulberry, BMul—black mulberry, WCur—white currant, RCur—red currant, BCur—black currant.

## Supplementary Materials

The following are available online at https://www.mdpi.com/2304-8158/8/12/646/s1, Table S1: Identification of phenolics in raspberry, grape, mulberry and currant extracts by DAD-ESI-MS.
