# Supplementary material for: The Relationship between Phytochemical Composition and Biological Activities of Differently Pigmented Varieties of Berry Fruits; Comparison between Embedded in Food Matrix and Isolated Anthocyanins
Source: Foods. 2019 Dec 5;8(12):646. doi: 10.3390/foods8120646 (PMC6963230; doi:10.3390/foods8120646)
Supplement: Supplementary file 1 [file foods-08-00646-s001.pdf]

Table 1S. Identification of phenolics in raspberry, grape, mulberry and currant extracts by DAD-ESI-MS

Peak no.	t_R [min]	λ_{max}(nm)	MS (+)	MS (-)	Mw	Tentative identification	Presence in extracts								
							YRas	RRas	WGra	RGra	WMul	BMul	WCur	RCur	BCur
1	3.2	240	215 (M+Na)	191 (M-H)	192	Quinic acid	+	+	-	-	-	-	-	-	-
2	4.6	270	171 (M+H)	169 (M-H)	170	Gallic acid	-	-	-	+	-	-	-	-	-
3	5.1	250	229 (M+Na)	205 (M-H)	206	nd	+	+	-	-	-	+	-	-	-
4	6.0	260	323 (M+Na)	299 (M-H)	300	Hydroxybenzoic acid hexoside	-	-	-	-	-	+	-	-	-
5	6.8	250, 300	132	198, 331	nd	nd	-	-	-	+	-	-	-	-	-
6	7.1	280	127	198, 523	nd	nd	-	-	-	+	-	-	-	-	-
7	7.2	260, 290sh	392 (M+Na)	368 (M-H)	369	nd	+	+	-	-	-	+	-	-	-
8	7.3	260	323 (M+Na)	299 (M-H)	300	Hydroxybenzoic acid hexoside	-	-	+	-	-	-	+	+	+
9	7.4	260	367 (M+Na)	343 (M-H)	344	nd	+	+	-	-	-	-	-	-	-
10	7.9	260, 290sh	635 (M+Na)	647 (M+Cl)	612	nd	-	-	-	-	-	+	-	-	-
11	8.5	260, 280sh, 325	-	353 (M-H)	354	Chlorogenic acid	-	-	-	-	-	-	-	-	+
12	8.7	280	291 (M+H)	279 (M-H)	290	(+)-Catechin	-	+	-	+	+	+	-	-	-
13	8.9	265, 295	353 (M+Na)	329 (M-H)	330	Vannilic acid hexoside	-	-	+	-	-	-	+	+	-
14	9	260, 330	618 (M+H)	616 (M-H)	617	nd	-	-	-	+	-	-	-	-	-
15	9.2	260, 290, 325	365 (M+Na)	341 (M-H)	342	Caffeic acid hexoside	+	+	-	-	-	+	+	+	-
16	10.0	280	579 (M+H)	577 (M-H)	578	Procyanidin dimer	-	-	-	+	-	+	-	-	-
17	10.2	300	349 (M+Na)	361 (M+Cl)	326	Coumaric acid hexoside	-	-	+	-	-	-	+	+	+
18	10.9	260,280sh	-	935 (M-H)	936	Galloyl bis-HHDP-hexoside	+	+	-	-	-	+	-	-	-
19	11.0	280	579 (M+H)	577 (M-H)	578	Procyanidin dimer	-	-	-	+	-	-	-	-	-
20	11.6	260, 300sh	467 (M+Na)	443 (M-H)	444	nd	-	-	-	+	-	-	-	-	-
21	11.7	300	349 (M+Na)	361 (M+Cl)	326	Coumaric acid hexoside	-	-	+	-	-	-	+	+	+
22	12.1	280	317	729	nd	nd	-	-	-	+	-	-	-	-	-
23	12.2	300	349 (M+Na)	361 (M+Cl)	326	Coumaric acid hexoside	-	-	+	-	-	-	+	+	+
24	12.4	300	349 (M+Na)	361 (M+Cl)	326	Coumaric acid hexoside	-	-	+	-	-	-	-	-	-
25	12.5	275	188, 205	-	nd	nd	-	-		+	-	-	+	+	+
26	12.9	260, 325	651 (M+Na)	627 (M-H)	628	nd	-	+	-	-	-	+	-	-	-
27	13.5	280	291	289	290	(-)-Epicatechin	-	-	-	+	-	+	-	-	-
28	13.9	275	338 (M+H)	372 (M+Cl)	337	nd	-	-	+	-	-	-	+	+	-
29	13.9	525	611 (M+H)	609 (M-H)	610	Cyanidin sophoroside	-	+	-	-	-	+	-	-	-
30	14.2	525	465 (M+H)	463 (M-H)	464	Delphinidin glucoside	-	-	-	+	-	-	-	-	+
31	14.4	525	757 (M+H)	755 (M-H)	756	Cyanidin rutinoside glucoside	-	+	-	-	-	+	-	-	-
32	14.9	525	611 (M+H)	609 (M-H)	610	Delphinidin rutinoside	-	-	-	-	-	-	-	-	+
33	15.0	525	581 (M+H)	579 (M-H)	580	Cyanidin sambubioside	-	-	-	-	-	-	-	+	-
34	15.2	525	727 (M+H)	725 (M-H)	726	Cyanidin xyloside rutinoside	-	-	-	-	-	-	-	+	-
35	15.3	525	449 (M+H)	447 (M-H)	448	Cyanidin glucoside	-	+	-	+	-	+	-	-	+
36	15.9	525	595 (M+H)	593 (M-H)	594	Cyanidin rutinoside	-	+	-	-	-	+	-	+	+
37	16.1	525	479 (M+H)	477 (M-H)	478	Petunidin glucoside	-	-	-	+	-	-	-	-	-
38	16.4	260, 360	619 (M+Na)	595 (M-H)	596	Quercetin arabinoside	-	+	-	-	-	-	-	-	-
39	16.7	525	271, 433	431, 467	432	Pelargonidin hexoside	-	-	-	-	-	+	-	-	-
40	16.9	260, 360	649 (M+Na)	625 (M-H)	626	Quercetin hexoside hexoside	-	-	-	-	-	+	-	-	-
41	17.0	260	418 (M+Na)	394 (M-H)	395	nd	-	-	-	-	-	-	+	+	+
42	17.2	250, 300sh, 360	435 (M+H)	433 (M-H)	434	Ellagic acid pentose conjugate	-	+	-	-	-	-	-	-	-
43	17.4	525	463 (M+H)	499 (M+Cl)	462	Peonidin glucoside	-	-	-	+	-	-	-	-	-
44	17.6	260, 350	487 (M+Na)	499 (M+Cl)	464	Quercetin hexoside	-	-	+	-	-	-	-	-	-

45	17.8	525	493 (M+H)	527 (M+Cl)	492	Malvidin glucoside	-	-	-	+	-	-	-	-	-
46	17.9	525	697 (M+H)	695 (M-H)	696	Cyanidin malonyl-diglucoside	-	-	-	-	-	+	-	-	-
47	18.1	260, 360	487 (M+Na)	499 (M+Cl)	464	Quercetin hexoside	-	-	+	-	-	-	-	-	-
48	18.2	260, 360	463 (M+H)	479 (M+Cl)	462	Isoramenthin hexoside	-	-	-	+	-	-	-	-	-
49	18.4	260, 360	503 (M+Na)	479 (M-H)	480	Myricetin glucoside	-	-	-	-	-	-	-	-	+
50	18.8	260, 360	633 (M+Na)	609 (M-H)	610	Quercetin galactoside rhamnoside	-	+	-	-	-	+	-	-	-
51	19.3	260, 300sh,360	597 (M+H)	595 (M-H)	596	Quercetin glucoside xyloside	-	-	-	-	-	+	-	-	-
52	19.8	525	303, 507	505	506	Delphinidin acetyl-glucoside	-	-	-	+	-	-	-	-	-
53	19.9	525	507, 773	771	772	Delphinidin coumaryl-diglucoside	-	-	-	+	-	-	-	-	-
54	20.0	250, 300sh, 360	435 (M+H)	433 (M-H)	434	Ellagic acid pentose conjugate	-	+	-	-	-	+	-	-	-
55	20.4	260, 360	479 (M+H)	477 (M-H)	478	Quercetin glucoronide	-	-	-	+	-	-	-	-	-
56	20.6	260, 360	633 (M+Na)	609 (M-H)	610	Rutin	-	-	+	-	-	-	+	+	+
57	20.7	260, 360	487 (M+Na)	463 (M-H)	464	Quercetin hexoside	-	-	-	+	-	+	-	-	+
58	21.1	525	787 (M+H)	821 (M+Cl)	786	Petunidin coumaryl-diglucoside	-	-	-	+	-	-	-	-	-
59	21.2	260, 360	529 (M+Na)	541 (M+Cl)	506	nd	-	-	-	-	-	-	+	+	+
60	21.4	260, 360	303 (M+H)	301 (M-H)	302	Quercetin	-	-	-	-	-	+	-	-	-
61	21.5	525	521 (M+H)	519 (M-H)	520	Petunidin acetyl-glucoside	-	-	-	+	-	-	-	-	-
62	21.7	250, 300sh, 360	435 (M+H)	433 (M-H)	434	Ellagic acid pentose conjugate	-	-	-	-	-	+	-	-	-
63	21.9	525	801 (M+H)	835 (M-H)	800	Malvidin coumaryl-diglucoside	-	-	-	+	-	-	-	-	-
64	22.5	260, 360	471 (M+Na)	447 (M-H)	448	Quercetin rhamnoside	-	-	-	-	-	-	-	+	-
65	22.6	250, 300sh, 360	449 (M+H)	447 (M-H)	448	Methyl ellagic acid pentose conjugate	-	+	-	-	-	-	-	-	-
66	22.7	525	535 (M+H)	569 (M+Cl)	534	Malvidin acetyl-glucoside	-	-	-	+	-	-	-	-	-
67	22.9	260, 310, 360sh	617 (M+Na)	593 (M-H)	594	Kaempferol coumaryl-glucoside	-	-	-	-	-	-	-	+	+
68	23.1	525	611 (M+H)	609 (M+Cl)	610	Delphinidin coumaryl-glucoside	-	-	-	+	-	-	-	-	-
69	23.3	260, 310	-	475 (M-H)	476	Ellagic acid acetyl-hexoside	-	+	-	-	-	-	-	-	-
70	23.4	525	611 (M+H)	609 (M-H)	610	Peonidin rutinoside	-	-	-	-	-	-	-	-	+
71	23.9	310	-	475 (M-H)	476	Ellagic acid acetyl-arabinoside	-	+	-	-	-	-	-	-	-
72	24.1	525	595 (M+H)	593 (M-H)	594	Cyanidin coumaryl-glucoside	-	-	-	-	-	-	-	-	+
73	24.1	525	625 (M+H)	623 (M-H)	624	Petunidin coumaryl-glucoside	-	-	-	+	-	-	-	-	-
74	24.6	525	639 (M+H)	673 (M+Cl)	638	Malvidin coumaryl-glucoside	-	-	-	+	-	-	-	-	-

- Hydroxbenzoic acid derivatives   - Hydroxycinnamic acid derivatives   - Flavan-3-ols   - Anthocyanins   -Flavonols
